# Tumor-Associated Macrophages as Major Immunosuppressive Cells in the Tumor Microenvironment

**DOI:** 10.3390/cancers16193410

**Published:** 2024-10-08

**Authors:** Anghesom Ghebremedhin, Dipti Athavale, Yanting Zhang, Xiaodan Yao, Curt Balch, Shumei Song

**Affiliations:** 1 Coriell Institute for Medical Research, 403 Haddon Ave., Camden, NJ 08103, USA; 2 Department Biomedical Sciences, Cooper Medical School of Rowan University, 401 Broadway, Camden, NJ 08103, USA; 3 MD Anderson Cancer Center at Cooper, Cooper University Hospital, 2 Cooper Plaza, Camden, NJ 08103, USA; 4 Departments of Surgery, Cooper University Hospital, 1 Cooper Plaza, Camden, NJ 08103, USA

**Keywords:** tumor-associated macrophages, immunosuppression, tumor microenvironment, cancers

## Abstract

**Simple Summary:**

The tumor microenvironment (TME) refers to the area immediately surrounding a cancerous tumor that influences the tumor’s behavior and how the immune system reacts to it. Central to the TME environment are special immune cells—tumor-associated macrophages (TAMs)—that, rather than restrict the tumor, actually facilitate tumor growth and metastasis. Thus, targeting these TAMs can help to eliminate cancer. In this article, we review the biology of TAMs and delve into the signaling mechanisms underlying the polarization of macrophage phenotypes, their plasticity, and therapeutic implications.

**Abstract:**

Within the tumor microenvironment, myeloid cells constitute a dynamic immune population characterized by a heterogeneous phenotype and diverse functional activities. In this review, we consider recent literature shedding light on the increasingly complex biology of M2-like immunosuppressive tumor-associated macrophages (TAMs), including their contribution to tumor cell invasion and metastasis, stromal remodeling (fibrosis and matrix degradation), and immune suppressive functions, in the tumor microenvironment (TME). This review also delves into the intricate signaling mechanisms underlying the polarization of diverse macrophage phenotypes, and their plasticity. We also review the development of promising therapeutic approaches to target these populations in cancers. The expanding knowledge of distinct subsets of immunosuppressive TAMs, and their contributions to tumorigenesis and metastasis, has sparked significant interest among researchers regarding the therapeutic potential of TAM depletion or phenotypic modulation.

## 1. Introduction

In 2022, global cancer data reported approximately 20 million new cancer cases, and 10 million deaths, worldwide. Demographics-based predictions suggest that new cancer cases will reach 35 million by 2050, underscoring the urgent need for advanced research, prevention strategies, and treatment options [1]. The increasing cancer burden calls for enhanced cancer research, new healthcare infrastructure, and public health initiatives. Key challenges, such as tumor heterogeneity and the complex, immunosuppressive tumor microenvironment (TME), contribute to poor treatment efficacy and poor patient outcomes [2]. Addressing these issues requires a multifaceted approach, including improved characterization of tumor heterogeneity, novel strategies to modify the TME, and personalized treatments that adapt to tumor dynamics.

The TME is a highly complex ecosystem, possessing heterogeneity between and within tumor types, as well as within individual tumors. Typically, the TME encompasses a spectrum of elements, including tumor cells, and a diverse array of immune cells, including lymphocytes (T cells and B cells), various myeloid cells (including macrophages), cancer-associated fibroblasts (CAFs), extracellular matrix components like collagen and fibronectin, and the tumor vasculature [3,4]. Recruitment of these cells to tissues is intricately regulated by specific receptors expressed by monocytes or macrophages, including colony-stimulating factor 1 (CSF1R) and Chemokine Receptor 2 (CCR2). Within the TME, tumor-associated macrophages (TAMs) are among the notable components, constituting approximately 50% of the tumor mass in most tumor types. TAMs, during the early stage of the tumor, are mostly anti-tumorigenic M1-like TAMs; these, however, are typically transformed into pro-tumorigenic M2-like TAMs as the tumor progresses. Hence, this review article focuses on the pro-tumorigenic M2-like TAM populations [5]. We summarize their roles in tumor initiation/progression, reshaping the TME, and suppressing anti-tumor immune responses. At the conclusion, we examine their possible therapeutic targeting.

## 2. Origin of Tumor-Associated Macrophages, and Their Recruitment to the TME

The majority of TAMs, within the TME, originate from circulating monocytes continually recruited to the tumor site. Cancer cells, TAMs, and other TME constituents (e.g., CAFs) are primary sources of chemoattractants, orchestrating monocyte recruitment [6,7,8]. Despite such recruitment of circulating monocytes, however, tissue-resident macrophages (TRMs) (arising during embryogenesis, in contrast to hematopoietic stem cells) also supplement the total pool of TAMs [9]. Following fetal development, these TRMs self-renew, into adulthood, protecting the local tissue [9]. Studies by Franklin et al. [10] revealed a progressive decrease in resident macrophage numbers, over time, in murine breast cancer models, paralleled by a concurrent increase in monocyte-derived TAMs. Notably, depletion of resident macrophages had no impact on tumor growth, whereas depletion of circulating monocytes led to improved outcomes [10]. Conversely, TRMs polarize into an M2 phenotype, replicating in a pancreatic ductal adenocarcinoma (PDAC) mouse model, favoring fibrotic desmoplasia. In this case, suppression of TRMs, but not circulating monocytes, regressed tumors [11].

TME plays a crucial role in shaping the composition and function of myeloid cells recruited from the circulation. Various chemokines, such as CCL2, CCL5, and CXCL12, orchestrate the recruitment of bone marrow-derived monocytes, which subsequently differentiate into mature macrophages upon arrival at the neoplastic site. This transition is facilitated by tumor-derived growth factors such as M-CSF and GM-CSF. Furthermore, tumor-produced factors (e.g., IL-4, IL-10, TGFß1, and PGE2) contribute significantly to the functional polarization of monocytes/macrophages into immunosuppressive cells, highlighting the intricate interplay between tumor cells and the immune system [12,13].

Phenotypic analysis of M2-like TAMs, by techniques such as single-cell RNA sequencing (scRNA-seq), flow cytometry, and immunohistochemistry, revealed the expression of various myeloid surface markers, including CD68, CD163, CD206, MGL, MARCO, and MS4A4A [14], as well as the immune checkpoint molecules PD-L1, TIM3, and VISTA [15,16,17,18]. These markers not only aid in defining and characterizing M2-like TAMs but also hold potential as prognostic indicators for cancer patients.

## 3. Cellular Signaling Involved in Macrophage Polarization in the TME

Traditionally, macrophages have been classified into M1 (classically activated) and M2 (alternatively activated) types. However, this classification is now considered too simplistic for macrophages in the TME, due to their considerable diversity, with additional macrophage subsets identified by scRNA-seq. Nonetheless, many TAMs exhibit characteristics more akin to M2-like macrophages, displaying immunosuppressive functions [19,20,21]. The polarization of macrophages within the TME toward either pro-tumorigenic/pro-fibrotic (M2) or anti-tumorigenic (M1) phenotypes involves intricate signaling pathways. Activation of the JNK signaling pathway drives macrophage polarization toward the M2 phenotype, whereas inhibiting JNK activity promotes the M1 phenotype. Cytokines such as IL-4, IL-10, and IL-13, under the regulation of signal transducer and activator of transcription-6 (STAT-6), induce M2-like macrophages by upregulating peroxisome proliferator-activated receptor (PPAR) expression [22,23]. Conversely, IFN-γ, a potent endogenous macrophage activator, triggers M1-like polarization through the IFN-γ/JAK/STAT-1 pathway [24]. AMP-activated protein kinase (AMPK) [25] and C/EBPb [26,27], further contribute to macrophage activation and polarization toward M2-specific gene expression.

Moreover, the involvement of interferon regulatory factors (IRFs) is notable, with IRF-1, IRF-5, and IRF-8 favoring M1-like polarization, while IRF-3 and IRF-4 promote the M2-like phenotype [28]. Activation of Akt1 and Notch induces an M1-like phenotype, whereas Akt2 activation promotes an M2-like phenotype [29,30]. These intricate signaling cascades underscore the dynamic interplay between macrophage polarization states and their impact on tumor progression. Macrophage polarization is also associated with significant metabolic dysregulation, including that of lactate, glutamine, succinate, and 2-ketoglutarate, affecting macrophage cellular pathways such as hypoxia, fatty acid oxidation, NF-κB, and epigenetic alterations [31]. Moreover, such differentially polarized macrophages demonstrate distinct gene signatures [32].

Besides via tumor cells themselves, macrophage polarization can also be influenced by various other cells in the tumor microenvironment (TME), including cancer-associated fibroblasts (CAFs), endothelial cells, platelets, adipocytes, and stellate cells. Signaling via the Hippo pathway, including its effectors YAP/TAZ, drives the expression/secretion of numerous cytokines (e.g., IL-4, IL-6, CSF-1) that help recruit monocytes to the TME, followed by their M2-like polarization into TAMs that express tumor immune checkpoints such as PD-1 [33].

## 4. Crosstalk between TAMs and Tumor Cells in the TME

TAMs play a pivotal role in tumor progression by serving as the primary source of CCL8 [34], whose interaction with SIGLEC1 orchestrates a positive feedback regulatory loop between tumor cells and TAMs, amplifying tumor cell motility. In response to cancer cell secretion of CCL8, the production of colony-stimulating factor-1 (CSF1), a critical factor supporting macrophage survival and proliferation, is stimulated, thus perpetuating the auto-stimulatory cycle. Moreover, heightened levels of CCL8 not only facilitate cancer–TAM communication but also serve as a potent monocyte chemoattractant, enhancing recruitment to the TME [34].

Several research studies highlight the infiltration of M2-like polarized TAMs into the delicate ecosystem of tumors, influencing tumor immunosuppression, cancer progression, angiogenesis, invasion, and metastasis. TAMs exert their pro-tumorigenic effects primarily through the secretion of a plethora of growth factors such as EGF, TGFβ, VEGF, and PDGFβ, which foster angiogenesis and tumor growth within the TME. As the principal source of these growth factors in the tumor milieu, TAMs significantly contribute to tumor development [35,36]. In neuroblastomas, it was also found that monocyte-derived macrophages and mesenchymal stem cells were recruited to closely colocalize at tumor sites, and then activated into TAMs and cancer-associated fibroblasts (CAFs), respectively [37].

In most human tumors, the presence of TAMs is intricately linked to unfavorable patient prognosis, as evidenced by numerous studies [38,39]. However, there exist intriguing exceptions to this paradigm, as observed notably in colorectal cancer [6,7,8,40,41,42]. TAMs contribute to tumor progression through multifaceted mechanisms, spanning from tumor initiation to metastasis, thereby influencing various hallmarks of cancer. Indeed, TAMs actively support the expansion of cancer stem cells by secreting a plethora of mediators, such as interleukin-6 (IL-6), platelet-derived growth factor (PDGF), milk fat globule-EGF factor 8 (MFG-E8), human cationic antimicrobial protein-18/LL-37 (hCAP-18/LL-37), and the recently implicated glycoprotein non-metastatic B (GPNMB) [43,44,45,46]. Additionally, cytokines derived from macrophages, notably interleukin-1 (IL-1), facilitate the recruitment and colonization of metastatic cancer cells at specialized niche sites [47,48,49]. TAMs, through their production of arginase-1, also metabolize arginine to ornithine and putrescine, which can promote tumor cell proliferation, while also suppressing cytotoxic T cells and downregulating the tumor-cytotoxin nitric oxide (NO) [50].

**Figure 1:**
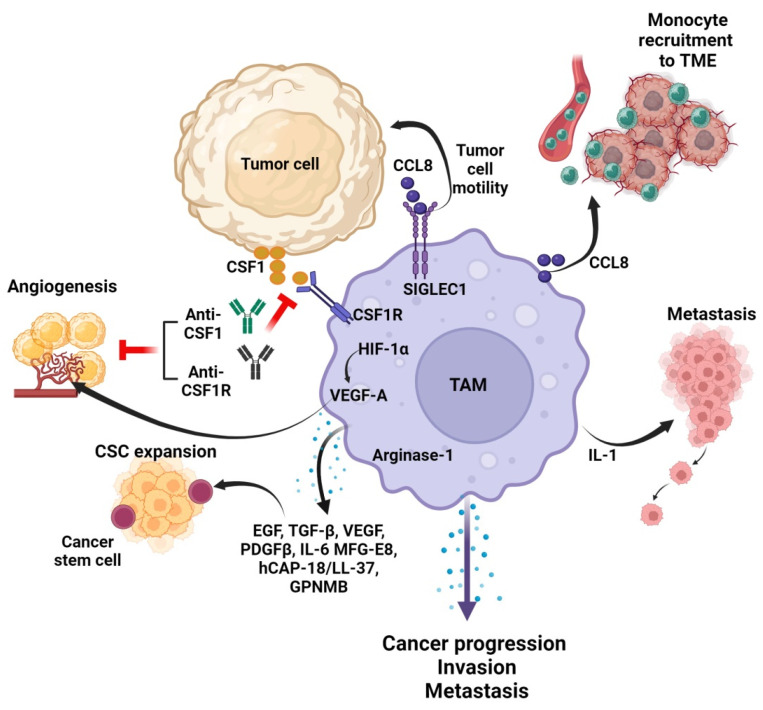
The interactions between TAMs and tumor cells in TME. TAMs govern tumor progression, metastasis, angiogenesis, invasion, and cancer stem cell (CSC) expansion via sections of various cytokines and growth factors. CCL8 produced by TAMs not only induces monocyte recruitment to TME but also promotes secretion of colony-stimulating factor-1 (CSF-1), which in turn supports TAM survival and proliferation. Abrogating the interaction between CSF1 and CSF1R inhibits tumor angiogenesis. CCL8 interacts with SIGLEC1 and promotes tumor cell motility. IL-1 supports the recruitment and colonization of metastatic cancer cells (Created in BioRender.com, accessed on 2 October 2024).

It was also found that in TAMs, hypoxia-inducible factor-1 (HIF-1α), during oxygen deprivation, activated vascular endothelial growth factor A (VEGF-A), involved in tumor blood vessel neogenesis [51]. Analogously, TAM levels were found to be associated with vascular density, in both translational models and human specimens [52]. Depletion of macrophages, via CSF1 inactivation, clodronate liposome administration, or CSF1 receptor (CSF1R) inhibition, has been linked to diminished angiogenesis across various preclinical models, including those employing MMTV-PyMT transgenic mice [53,54]. Furthermore, TAMs can directly impede the efficacy of anti-tumor drugs through various mechanisms, including the production of protein-degrading enzymes or sequestration of therapeutic agents, thereby obstructing their intended targets. This multifaceted role of TAMs underscores their significance in shaping the TME while highlighting the challenges of developing effective therapeutic strategies against cancer. Furthermore, TAMs contribute to cancer cell invasion and metastatic progression by secreting proteolytic enzymes that facilitate extracellular matrix (ECM) digestion [55]. Figure 1 demonstrates tumor cell and TAM interactions and their downstream effects.

## 5. Immunosuppressive Functions of TAMs in the TME

Repression of tumor immunity, within the TME, can also occur via tumor cell secretion of paracrine-acting factors (e.g., IL-10 and PGE2), resulting in polarization of monocytic MDSCs into M2-like TAMs; analogously, inhibition of prostaglandin synthesis (via COX2 inhibition) reversed this effect [12,13]. Additionally, IL-1 has been implicated in driving the upregulation of TET2 expression, a DNA methylcytosine dioxygenase, thereby sustaining TAM-mediated immunosuppression in both murine and human melanoma [56]. Macrophages play a crucial role in regulating immune responses, and they produce several inhibitory molecules that can modulate the activity of T cell lymphocytes. M2-like TAMs have upregulated Programmed Death-Ligand 1 (PD-L1) ligand, a ligand that interacts with PD-1 on T cells, leading to T cell exhaustion and reduced immune responses against tumors [57,58,59]. M2-like TAMs also express Fas Ligand (FasL) which binds to Fas on T cells, inducing apoptosis and promoting immune tolerance [60]. Furthermore, M2-like TAMs upregulate HLA-G, a non-classical MHC molecule that can inhibit natural killer (NK) cell activity and T cell responses, contributing to immune tolerance [61].

Moreover, myelomonocytic cells induce metabolic starvation in T cells through arginase activity and the production of amino acid metabolites, via indoleamine 2, 3-dioxygenase (IDO1). IDO1 catalyzes the degradation of tryptophan (Trp), along the kynurenine pathway, and is implicated in the induction and expansion of Treg populations [62]. Moreover, M2-like macrophages express various immune checkpoint proteins (e.g., B7-H4, PD-L1, VISTA), resulting in immunosuppression; various neutralizing antibodies were found to reverse this effect [6,63,64,65].

**Figure 2:**
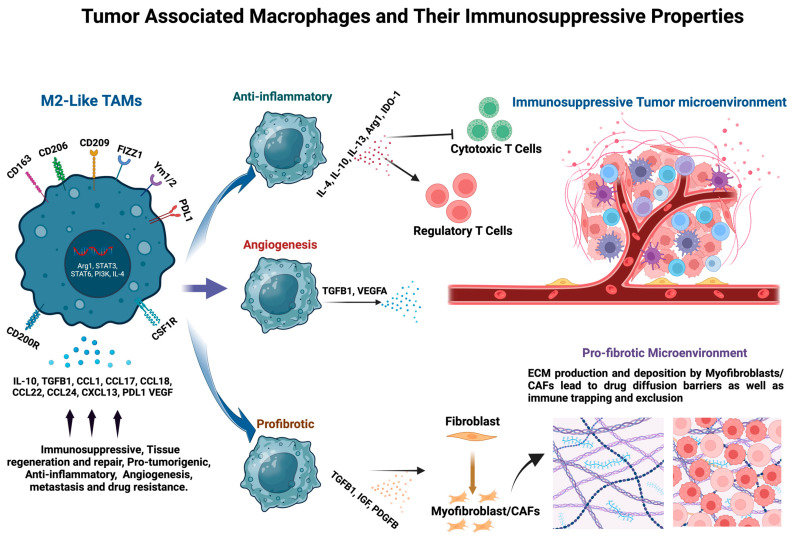
M2-like TAMs and Their Pro-tumorigenic Characteristics: TAMs are among the most prevalent cell types present in the tumor microenvironment (TME). They are crucial in shaping and sustaining a pro-tumorigenic TME by suppressing inflammatory immune responses, facilitating new blood vessel growth, and fostering a fibrotic microenvironment. These activities of TAMs contribute to tumor progression, resistance to therapy, and tumor dissemination to distant sites (Created in BioRender.com, accessed on 2 October 2024).

TAMs also play a pivotal role in promoting T cell apoptosis and immunosuppression, through the secretion of IL-10 and TGF-β1, thereby hindering the body’s natural defense mechanisms against cancer cells (Figure 2) [66]. TAM secretion of IL-10 impedes dendritic cells from activating anti-tumor T cell responses, while TGF-β1 production by TAMs fosters the survival of regulatory T cells, further exacerbating the immunosuppressive environment. In a murine model of multiple myeloma (MM), T cell activity was suppressed by infiltration of myeloid-derived suppressor cells (MDSCs), as facilitated by TAM-secreted IL-1β and IL-18 [67]. Analogously, Il-18 knockout mice forestalled MM progression, via CD8+ T cell activity, and bone marrow IL-18 levels correlated negatively with survival, in 73 MM patients [67].

## 6. Stromal Remodeling by TAMs in the TME

Fibrosis plays a well-documented role in the development and onset of various cancers, including pancreatic and liver cancers. In pancreatic ductal adenocarcinoma (PDAC), a serious fibrotic reaction, known as desmoplasia, is a prominent feature. The desmoplastic stroma is rich in activated pancreatic stellate cells (PSCs) and extracellular matrix (ECM), and plays a significant role in tumor initiation as well as progression [68,69]. The role of fibrosis in cancer is also well illustrated by liver cancer, with both hepatocellular and cholangiocarcinoma developing in cirrhotic and fibrotic livers, and fibrosis contributing to immunosuppressive pathways and cascades activated by tissue stiffness [70]. In addition to their pro-tumorigenic roles in the tumor microenvironment, M2-like macrophages also promote tissue fibrosis by secreting the profibrotic cytokine TGFβ1 to induce the differentiation of resident fibroblasts to effector myofibroblasts, which can produce a variety of collagens that promote wound healing. In addition to TGF-β, vascular endothelial growth factor (VEGF), platelet-derived growth factor (PDGF), and angiotensin activate stromal cells such as myofibroblasts, resulting in excessive deposition of ECM proteins [71].

TAMs also exert their influence indirectly by affecting various cell types, or the ECM, within the TME. Activated by type II cytokines like IL-4, IL-10, or IL-13, as well as other environmental cues, M2-like TAMs contribute to tissue repair, remodeling, and fibrosis [72]. Thus, TAMs are intimately involved in stromal remodeling. For example, TAMs destroy ECM components, via the release of metalloproteinases, cathepsins, and urokinase plasminogen activator (UPA), releasing matrix-bound growth factors and immunosuppressive cytokines [73], while also depositing neoplastic ECM constituents (Figure 2) such as osteopontin, tenascin, fibronectin, and SPARC (secreted protein acidic and rich in cysteine) [74]. These cumulative activities attenuate extracellular barriers and create a favorable environment for tumor cell migration and invasion.

Notably, in certain cancers such as early pancreatic adenocarcinoma [11] and colon cancer [75], macrophages directly foster fibrosis by ECM deposition. M2 macrophages also highly express PI3Kγ, a signaling molecule that promotes tissue fibrosis by promoting M2 polarization of macrophages. PI3Kγ, also expressed in endothelial cells and fibroblasts [76,77,78,79], likely elicits tumor infiltration of leukocytes, as PI3Kγ-deficient rodents had extended survival and attenuation of bleomycin-induced fibrosis compared to PI3Kγ-wild-type rodents. In parallel, PI3Kγ-knockout mice upregulated IFN-γ and IL-10, while downregulating TGFβ1, CCL2, collagen, fibronectin, and α-SMA, in addition to having less leukocyte ingress, following bleomycin challenge [80]. In turn, cancer-associated fibroblasts (CAFs) release numerous factors (e.g., periostin, FNT, IGF, and exosomal microRNAs) that promote tumor progression, chemoresistance, and gene amplification [37].

## 7. Current Strategies to Target Immunosuppressive TAMs

Current macrophage-targeting therapies hold significant promise for treating cancers. Macrophages play pivotal roles in both innate and adaptive immunity, making them attractive targets for therapeutic intervention. In cancer, tumor-associated macrophages (TAMs) often promote tumor growth, angiogenesis, immunosuppression, and metastasis, presenting opportunities for targeted therapies to modulate their functions [8] (Figure 2). Therapeutic strategies directed at TAMs can be grouped into four areas: limiting monocyte recruitment, targeting TAM activation, reprogramming TAMs to anti-tumor macrophages, and targeting TAMs in combination with standard therapies.

Strategies aimed at limiting monocyte recruitment involve targeting recruitment signals. This can be achieved by inhibiting the expression or activity of chemokines such as CCL2 (also known as MCP-1) or CSF-1 (colony-stimulating factor 1), crucial for monocyte recruitment [8,41]. Additionally, blocking adhesion molecules such as integrins or selectins that facilitate the migration of monocytes into the TME can impede TAM infiltration. Once recruited to the TME, monocytes differentiate into TAMs under the influence of various stimuli, such as cytokines (e.g., IL-4, IL-10, TGF-β) and signaling pathways (e.g., STAT3, NF-κB). Therapeutic strategies targeting TAM activation and differentiation aim to interrupt these signaling cascades or inhibit the receptors involved in TAM polarization. For instance, blocking receptors such as CSF-1R (colony-stimulating factor 1 receptor) or TLRs (Toll-like receptors) can prevent TAM activation and polarization toward a pro-tumoral phenotype [81].

TAMs exhibit plasticity and can be reprogrammed from a pro-tumoral M2-like phenotype to an anti-tumoral M1-like phenotype, representing the most common therapeutic approach. This can be achieved through various means, including the administration of cytokines (e.g., IFN-γ, TNF-α) or agents that activate pattern recognition receptors (PRRs), to induce M1-like polarization. Additionally, targeting specific metabolic pathways or epigenetic modifications involved in TAM polarization can promote their reprogramming toward an anti-tumoral phenotype. For example, CD206, also known as the macrophage mannose receptor, has also emerged as a promising target for cancer therapies, due to its overexpression on TAMs, in various cancers. By targeting CD206, researchers aim to modulate TAM activity, reprogramming them from a pro-tumoral M2-like phenotype to an anti-tumoral M1-like phenotype, thus enhancing anti-tumor immune responses [82,83]. Several preclinical studies have demonstrated the efficacy of CD206-targeting therapies in inhibiting tumor growth, and improving overall survival, in various cancer models [84,85]. However, the clinical translation of these findings is still in its early stages, with ongoing efforts focused on developing safe and effective CD206-targeting agents (including nanoscale cargos) for cancer treatment [86,87].

TAM-targeted therapies are often combined with standard treatment modalities such as chemotherapy, radiotherapy, or immunotherapy, to enhance their efficacy. Combinatorial approaches aim to exploit synergistic effects between TAM-targeted therapies and standard treatments, to overcome tumor resistance mechanisms and improve treatment outcomes. For example, combining TAM-targeted agents with immune checkpoint inhibitors can enhance the anti-tumor immune response by reprogramming TAMs, and overcoming immunosuppression, within the TME. In the syngeneic Lewis lung carcinoma mouse, it was found that blockade of a subset of TAMs expressing the immune checkpoints TIM3 and VISTA resensitized tumors to paclitaxel [18]. Moreover, small molecule inhibitors targeting intracellular signaling pathways in myeloid cells, such as Janus kinase (JAK) inhibitors, have demonstrated efficacy in various inflammatory conditions and cancers [88,89]. A list of current TAM-targeting pharmacological agents is provided in Table 1 below.

## 8. Conclusions and Future Perspectives

Recent investigations into the intricate interplay between tumor-associated macrophages (TAMs) and malignant tumors have underscored TAMs’ emerging role as promising biomarkers for cancer diagnosis, prognosis, and therapeutic intervention. In human studies, TAMs are frequently characterized by elevated levels of CD163, CD206, and CD204 expression. While these markers lack specificity for individual cancer types, their heightened expression typically correlates with unfavorable clinical outcomes.

The crucial involvement of TAMs in promoting cancer progression and metastasis emphasizes their critical role as primary targets for therapeutic intervention in cancer management. Due to the complex interplay between cancer cells and TAMs, focusing solely on one signaling pathway may prove insufficient. Thus, the pursuit of effective therapies targeting TAMs requires a sophisticated approach, to understand the multitude of factors influencing macrophage polarization. A thorough investigation and characterization of TAM characteristics, across different cancer types, stages, and tissue environments, is essential for the development of potent strategies aimed at effectively targeting this unique cell type.

## Figures and Tables

**Table 1:** List of pharmacological inhibitors targeting M2-like TAMs.

Inhibitors	Mechanism of Action	References
Anti-CSF1R	Blocks CSF1R signaling, depleting M2-like TAMs	[84,90]
STAT6 Inhibitors	Blocks STAT6 signaling pathway involved in M2-like TAMs polarization	[91,92]
STAT3 Inhibitors	Blocks STAT3 signaling pathway involved in M2-like TAMs polarization	[93,94]
Anti CD47-SIRPa	Promotes TAMs phagocytic activity	[95,96,97,98]
Anti-CD24/Siglec-10	Inhibit the CD24/Siglec-10 signaling pathway, Promote TAMs phagocytic activity	[99,100]
Anti-PDL1	Blocks PD1-PDL1 interaction, leading to T Cell activation and boosted immune response	[57,58,59]
Chlodronate liposomes	Depletes tumor-associated macrophages	[101,102]
IDO Inhibitors	Inhibit indoleamine 2,3-dioxygenase, altering macrophage polarization	[103,104]
PI3Kγ-Inhibitors	Inhibit myeloid cell recruitment and promote M1-like phenotype	[105]
CD40 agonists	Activate CD40 and promote M1 macrophage phenotype	[106,107]
Anti-CCL2/CCR2	Targets CCL2/CCR2 axis, Inhibits TAMs recruitment	[108,109]
Anti-CCL5/CCR5	Inhibits TAMs recruitment	[110,111,112]
